# Adiponectin improves amyloid‐β 31‐35‐induced circadian rhythm disorder in mice

**DOI:** 10.1111/jcmm.16932

**Published:** 2021-09-15

**Authors:** Yuan Yuan, Chen Li, Shuai Guo, Cong Sun, Na Ning, Haihu Hao, Li Wang, Yunfei Bian, Huirong Liu, Xiaohui Wang

**Affiliations:** ^1^ Basic Medical Sciences Center Shanxi Medical University Taiyuan China; ^2^ Key Laboratory of Cellular Physiology (Shanxi Medical University) Ministry of Education Taiyuan China; ^3^ Department of Orthopedics Shanxi Bethune Hospital & Shanxi Academy of Medical Sciences Taiyuan China; ^4^ Department of Pathology Shanxi Medical University Taiyuan China; ^5^ Department of Cardiology The Second Hospital of Shanxi Medical University Taiyuan China; ^6^ Department of Physiology and Pathophysiology, School of Basic Medical Sciences Capital Medical University Beijing China

**Keywords:** adiponectin, Alzheimer's disease, Aβ31‐35, Bmal1, GSK3β

## Abstract

Adiponectin is an adipocyte‐derived hormone, which is closely associated with the development of Alzheimer's disease (AD) and has potential preventive and therapeutic significance. In the present study, we explored the relationship between adiponectin and circadian rhythm disorder in AD, the effect of adiponectin on the abnormal expression of Bmal1 mRNA/protein induced by amyloid‐β protein 31‐35 (Aβ31‐35), and the underlying mechanism of action. We found that adiponectin‐knockout mice exhibited amyloid‐β deposition, circadian rhythm disorders and abnormal expression of Bmal1. Adiponectin ameliorated the abnormal expression of the Bmal1 mRNA/protein caused by Aβ31‐35 by inhibiting the activity of glycogen synthase kinase 3β (GSK3β). These results suggest that adiponectin deficiency could induce circadian rhythm disorders and abnormal expression of the Bmal1 mRNA/protein, whilst exogenous administration of adiponectin may improve Aβ31‐35‐induced abnormal expression of Bmal1 by inhibiting the activity of GSK3β, thus providing a novel idea for the treatment of AD.

## 1 INTRODUCTION

Since the 21st century, Alzheimer's disease (AD) has become a serious health problem that affects ageing populations worldwide. Circadian rhythm disorder occurs in the early stage of AD, and this can induce impairment of learning and memory in AD.^1^, ^2^ Therefore, circadian rhythm disorders play a vital role in the development of AD. Further studies have found that circadian rhythm disorders are closely related to the extracellular aggregates of amyloid‐β protein (Aβ) in the brain, which plays a causative role in AD pathogenesis.^3^ Our previous study found that the intrahippocampal injection of amyloid‐β protein 31‐35 (Aβ31‐35) resulted in circadian rhythm disorder in C57BL/6 mice.^4^

Circadian rhythms are rhythmic oscillations that spontaneously form in organisms. Their maintenance depends on the transcriptional‐translational feedback loop composed of a series of clock genes and proteins, amongst that Bmal1 is an important positive regulator.^5^ Studies have found that Bmal1^−/−^ mice lose circadian rhythmicity at the behavioural and molecular levels,^6^ and the triple‐transgenic AD mouse model exhibits Aβ deposition in the brain and abnormal expression of *Bmal1*.^7^ Our previous study found that Aβ31‐35 induces abnormal expression of Bmal1 mRNA/protein in HT22 cells.^8^ However, there is still no effective measure to reverse the Aβ31‐35‐induced abnormal expression of Bmal1, and the underlying mechanism is not yet clear.

Studies have shown that AD is closely related to type 2 diabetes mellitus (T2DM) in pathogenesis. Insulin resistance is a common pathophysiological characteristic of these two diseases and plays a significant role in the development of AD. Adiponectin (APN) is an adipocytokine secreted mainly by adipocytes, with insulin‐sensitizing effects via the activation of insulin signalling pathways.^9^ Clinical studies have shown that circulating adiponectin levels are decreased in patients with mild cognitive impairment and AD.^10^ Recent studies have also found that APN signal transduction defects are sufficient to induce AD‐like phenotypes in mice, including Aβ deposition, tau protein hyperphosphorylation, synaptic loss and neuronal apoptosis.^11^, ^12^ APN can enhance insulin sensitivity in SH‐SY5Y cells by activating AdipoR1 and APN signalling to alleviate neuropathological deficits and clinical manifestations in APP/PS1 mice, such as Aβ aggregation, synapse dysfunction, memory and cognitive deficits.^11^, ^13^ These results reveal that APN is closely associated with the development of AD and has potential preventive and therapeutic significance for AD. In contrast, the role of APN in AD circadian rhythm disorder remains uncertain, and whether APN can improve the abnormal expression of Bmal1 mRNA/protein caused by Aβ31‐35 has not been documented.

Adiponectin has also been reported to regulate insulin sensitivity to activate insulin signalling and inhibit glycogen synthase kinase 3β (GSK3β) activity.^11^ GSK3β is a serine‐threonine kinase involved in the regulation of circadian rhythm,^14^ and it is closely related to the regulation of Bmal1. Studies have shown that GSK3β can directly phosphorylate and degrade BMAL1.^15^ Studies have revealed that abnormal deposition of Aβ can increase the activity of GSK3β.^16^ However, whether GSK3β activation induced by Aβ affects the expression of Bmal1 mRNA/protein and whether APN can improve the abnormal expression of Bmal1 induced by Aβ31‐35 by inhibiting the activity of GSK3β are still unclear. This study explored the relationship between APN and circadian rhythm disorder in AD and the effect of APN on Aβ31‐35‐induced abnormal expression of Bmal1 mRNA/protein and its possible mechanism.

## 2 MATERIALS AND METHODS

### 2.1 Experimental animals

All experimental procedures were approved by the Ethics Committee of Shanxi Medical University. All experiments were performed in accordance with the guidelines of the National Institutes of Health Guide for the Care and Use of Laboratory Animals. The Experimental Animal Center of Shanxi Medical University provided 4‐month‐old (10–20 g) and 12‐month‐old (25–35 g) male C57BL/6 mice, and 4‐month‐old and 12‐month‐old global adiponectin‐knockout (APN‐KO) mice were purchased from the Shanghai Model Organisms Center, Inc. The APN‐KO mouse model was generated by the homologous recombination method. These APN^+/−^ mice (between heterozygotes) were mated and reproduced to obtain three genotype mice of wild‐type, heterozygous and homozygous. Genotyping was employed for the identification of homozygous adiponectin‐knockout mice (APN^−/−^ mice). The mice with diet ad libitum were kept in a suitable environment with a room temperature of 20–24°C and humidity of 35%–55%. The use of animals in the experiments in this study was in accordance with the National Experimental Animal Use Regulations.

### 2.2 Polymerase chain reaction

Polymerase chain reaction (PCR) was used to confirm the genotype of the experimental mice. Tail tissues (approximately 0.5 cm) were digested overnight in 500 μl lysis buffer containing 5 μl proteinase K. The digested tissue was added to 500 μl of phenol/chloroform mixed solution (equal volume) and centrifuged at 4°C and 13523*g* for 15 min. The supernatant (200 μl) was mixed with 400 μl of absolute ethanol (double volume of absolute ethanol) to precipitate the DNA. The white flocculent DNA was washed twice with 70% ethanol and centrifuged to discard the supernatant. The pelleted DNA was air‐dried and dissolved in 100 μl of Tris‐EDTA buffer. The extracted DNA was amplified. The APN‐KO mouse model was generated by the homologous recombination method. Exons 2 and 3 of the APN gene were replaced with the pGK‐neo gene and three primers were designed according to the insertion position. The APN‐KO mouse primers were P1 (GGCTCTCTGGGAGAGGCGAGT), P2 (CCATCACGGCCTGGTGTGCC) and P3 (TTCGCCATTCAGGCTGCGCA). The PCR‐amplified DNA was detected by agarose gel electrophoresis at a constant voltage of 110 V and visualized by bromophenol blue staining. Compared with those of the DNA marker, the observed DNA bands were 326 bp for wild‐type mice and 531 bp for homozygous APN‐KO, and PCR products of heterozygous mice had two DNA bands located at 531 bp and 326 bp (Figure S1).

### 2.3 HT22 cell culture

**FIGURE 1:**
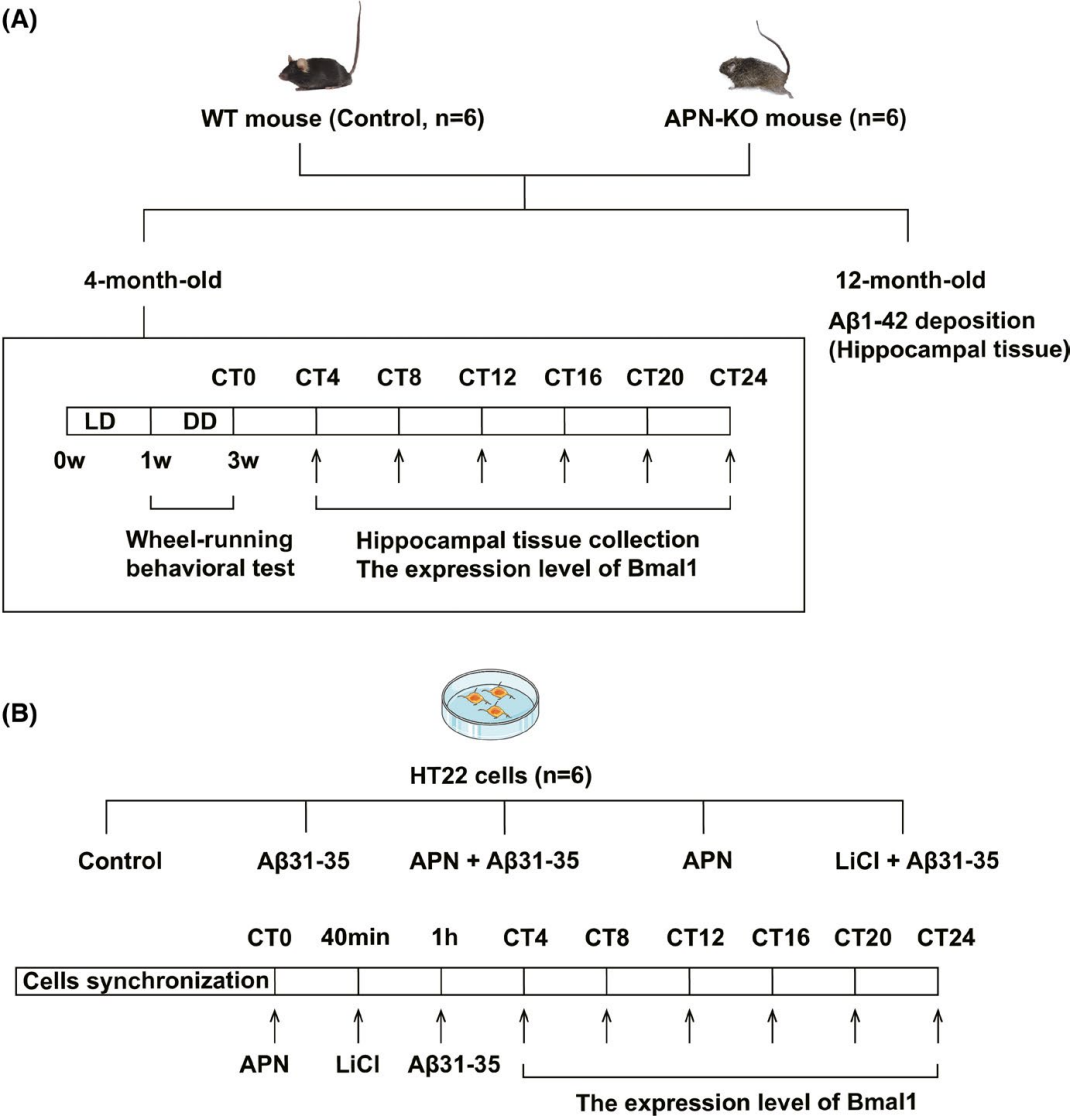
Timelines of experiments of animal model and cell model. (A) The timeline of experiments of animal model. (B) The timeline of experiments of cell model

The mouse hippocampal nerve cell line (HT22) were purchased from Guangzhou Jennio Biotechnology Co., Ltd. The HT22 cells were cultured in Dulbecco's Modified Eagle Medium complete medium (HyClone) supplemented with 10% foetal bovine serum (FBS; Sciencell) and were kept in a constant‐temperature incubator at 37°C and 5% CO_2_. After adhesion, cells with 80% density and adequate growth conditions were selected for the synchronization treatment in each group. The complete medium for culturing cells was replaced with a starvation medium supplemented with 1% FBS. HT22 cells were synchronized by serum deprivation (1% FBS) for 1 h, which was regarded as circadian time 0 (CT0), and then treated with the complete medium again. The synchronized cells were cultured for *n* hours, denoted as CTn.^17^ Next, different treatments were performed on synchronized cells. The control group cells were cultured in the complete medium. The cells of the Aβ31‐35 group were treated with 5 μmol/L Aβ31‐35 (Abcam). The cells in the APN +Aβ31‐35 group were first pretreated with 10 ng/ml APN (Pepro Tech)for 1 h and then treated with 5 μmol/L Aβ31‐35. The cells in the APN group were treated with 10 ng/ml APN alone. In the LiCl (Sigma) + Aβ31‐35 group, the cells were pretreated with 30 μmol/L LiCl (a specific inhibitor of GSK3β^18^) for 20 min, followed by the addition of 5 μmol/L Aβ31‐35 (Figure 1B).

### 2.4 Immunohistochemical staining

The full‐length Aβ1‐42 is more neurotoxic and immunohistochemical staining was used to detect Aβ1‐42 deposition in 12‐month‐old APN‐KO mice and C57BL/6 mice. The brain tissue located 4 mm behind the optic chiasm was fixed in 4% paraformaldehyde, embedded in paraffin wax after 24–48 h and sectioned at a thickness of approximately 5 mm. The sections were dehydrated and then treated with 3% H_2_O_2_ in the dark for 15 min, followed by antigen retrieval with EDTA buffer. The sections were blocked with 10% goat serum at room temperature for 10 min and incubated with the primary antibody (anti‐Aβ1‐42, concentration 1:1200; Abcam) at 4°C overnight. The sections were then incubated with the secondary antibody at 37°C for 45 min. Brown colour staining was developed with diaminobenzidine (DAB) chromogenic solution. The sections were counterstained with haematoxylin, differentiated with 1% hydrochloric acid alcohol, and sealed with neutral resin. An Olympus optical micrograph system was used for image acquisition.

### 2.5 Wheel‐running behavioural test

The circadian rhythm of each group of male mice (*n* = 6) was evaluated using a wheel‐running behavioural test. Power analysis was performed to evaluate the sample size for behavioural animal experiments. The 4‐month‐old APN‐KO mice and C57BL/6 mice were placed in a well‐ventilated running wheel device at a temperature of 22 ± 2°C and humidity of 35%–55%. The lighting environment was set to 12 h of light and 12 h of darkness (Light‐Dark, LD) for 1 week; that is, the lights were turned on at 6:00 and turned off at 18:00. Then, the environment was changed to constant darkness for 2 weeks. Due to the lack of light, the endogenous biological rhythms of the animals were represented by circadian time (CT).^19^ The length of each circadian cycle was divided into 24 equal parts, and each part was 1 CT. The time at which the mice started their daily activities was defined as CT12.^4^ The running wheel activity was recorded using the VitalView system at a frequency of every 5 min. The running wheel data were analysed using ActiView software, accompanied by the acquisition of the original map of the wheel‐running activity, free‐running cycle and day and night activities. Upon the termination of wheel‐running, the mice were decapitated at CT4, CT8, CT12, CT16, CT20 and CT24, and the hippocampal tissue was peeled off on ice to further detect the expression of Bmal1 mRNA/protein (Figure 1A).

### 2.6 Real‐time PCR

*Bmal1* mRNA expression levels were detected using real‐time PCR at different CT points. Total RNA from mouse hippocampus and HT22 cells was extracted by the Trizol method and reverse transcribed to cDNA and then specifically amplified using the SYBR Green kit. The corresponding primer design was as follows: *Bmal1* (Gen‐Bank ID NM_001243048.1), forward: 5′‐ACGACATAGGACACCTCGCAGA‐3′, reverse: 5′‐TCCTTGGTCCACGGGTTCA‐3′; glyceraldehyde‐ 3‐phosphate dehydrogenase (GAPDH) (Gen‐Bank ID NM_008084.2), forward: 5′‐AAATGGTGAAGGTCGGTGTGAAC‐3′, reverse: 5′‐CAACAATCTCCACTTTGCCACTG‐3′. All data were standardized with the expression of GAPDH at CT4 in the control group, and the target gene mRNA was quantified using the 2^−ΔΔ^ *^C^* ^t^ method.

### 2.7 Western blotting

The expression of BMAL1 protein was detected by western blotting. The mouse hippocampal and HT22 cells were lysed on ice for 1.5 h with RIPA lysis buffer, and the supernatant was extracted after centrifugation at 13523*g* for 15 min. The protein concentration was quantified using the bicinchoninic acid (BCA) method, and the protein content was quantified as 40 μg. The protein was completely denatured after adding 5 × loading buffer and heating at 100°C for 10 min. The protein samples were subjected to SDS‐PAGE and then transferred to a polyvinylidene fluoride membrane. The membrane was blocked with 5% skimmed milk at room temperature for 2 h and then incubated with primary antibodies against BMAL1 (Abcam), pGSK3β^S9^ (BBI), GSK3β (BBI), β‐actin and GAPDH overnight at 4°C. After washing with Tris Buffered Saline with Tween (TBST), the membrane was incubated with the corresponding secondary antibody for 2 h at room temperature, followed by washing with TBST again. Images were exposed and captured using a gel imaging system. Western blot data were quantified using ImageJ software.

### 2.8 Statistical analysis

Statistical analysis was performed using SPSS software (version 16.0). The normal distribution of measured data was presented as group mean ± standard error of mean. Statistical analyses were performed using one‐way analysis of variance for multiple group comparisons and a least significant difference *t* test for comparison between groups. The results were presented as *α* = 0.05, and statistical significance was set at *p* < 0.05.

## 3 RESULTS

### 3.1 Adiponectin‐knockout mice exhibited Aβ deposition and circadian rhythm disorders

**FIGURE 2:**
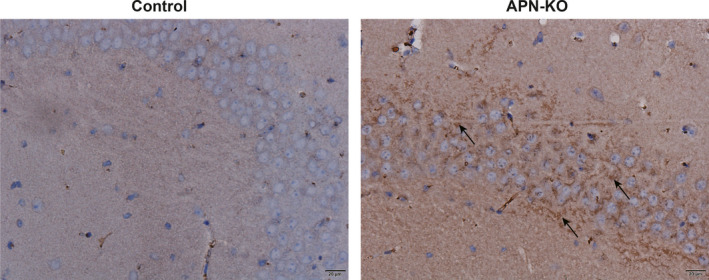
Abnormal deposition of Aβ1‐42 in the hippocampus of APN‐KO mice (*n* = 6, Scale bar: 20 µm)

To explore the correlation between APN and AD, we first used immunohistochemical staining to detect Aβ1‐42 deposition in the hippocampus of 12‐month‐old C57BL/6 mice and APN‐KO mice. An aggregation of brownish‐yellow granules was observed in the hippocampus of APN‐KO mice when compared to C57BL/6 mice (Figure 2), suggesting that APN deficiency could induce abnormal Aβ deposition in the hippocampus.

**FIGURE 3:**
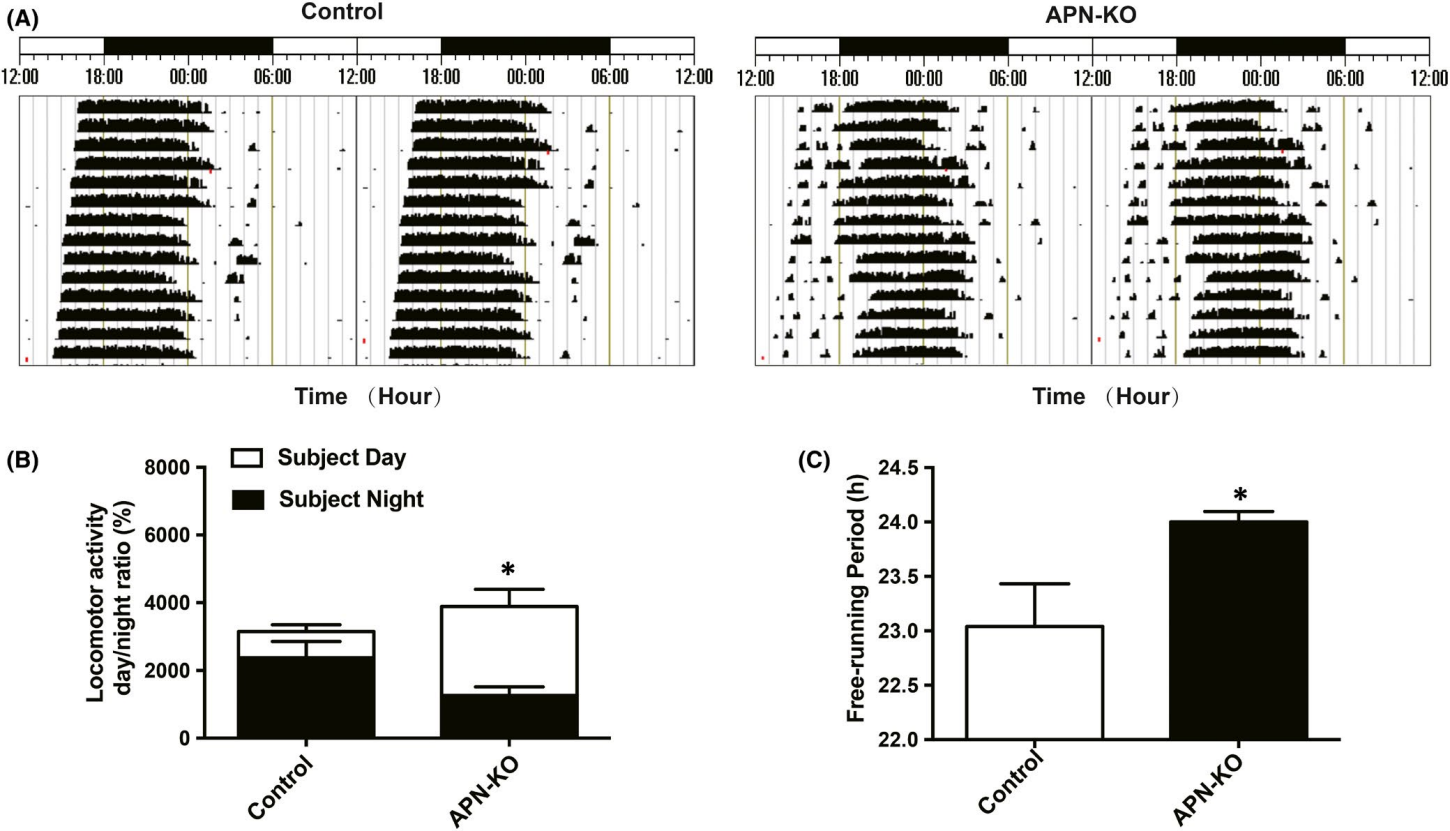
APN‐KO mice exhibited circadian rhythm disorders. (A) Representative locomotor activity records of each group. (B) The ratio of subjective day and night locomotor activity to total locomotor activity in each group. (C) The free‐running period of the locomotor activity rhythm in each group. Data are expressed as mean ± standard error of mean (SEM) (*n* = 6 per group). *p < 0.05 compared with the control group

Subsequently, we selected 4‐month‐old C57BL/6 mice and APN‐KO mice to conduct wheel‐running experiments to clarify the effect of APN deficiency on circadian rhythm. The results showed that the mice in the control group displayed rhythmic wheel‐running activity with clearly demarcated movement and rest phases. The activities mainly occurred during subjective nights, and the starting time was relatively fixed (Figure 3A). The ratio of subjective daytime activity to total activity was 27.34 ± 9.36%, and the free‐running period was 23.04 ± 0.39 h (Figure 3B,C). Conversely, APN‐KO mice displayed circadian rhythm disorder that was manifested by changes in the starting time of daily activities, increased subjective daytime activities and reduced subjective night activities (Figure 3A). The ratio of subjective daytime activity to total activity increased significantly (Figure 3B), and the free‐running period was prolonged (*p* < 0.05) (Figure 3C). Collectively, these results showed that APN deficiency could induce circadian rhythm disorders in C57BL/6 mice.

### 3.2 Abnormal expression of Bmal1 mRNA/protein in the hippocampus of APN‐KO mice

**FIGURE 4:**
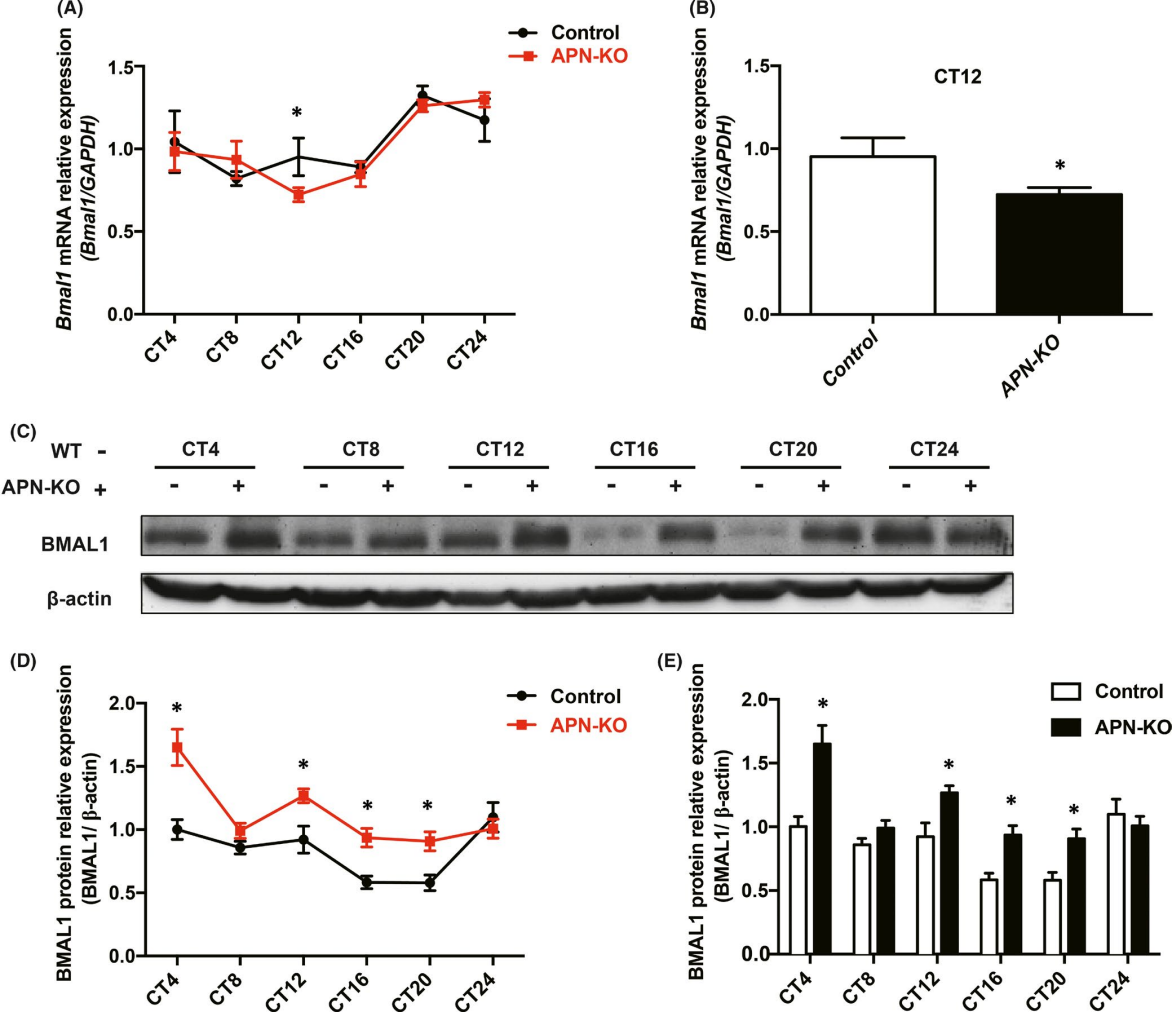
Abnormal expression of Bmal1 mRNA/protein in the hippocampus of APN‐KO mice. (A) mRNA expression of *Bmal1* in the hippocampal tissue at different time points. (B) mRNA levels of *Bmal1* at CT12 in each group. (C) The expression levels of BMAL1 protein in the hippocampus at different time points. (D) Broken line chart of BMAL1 protein expression at different time points. (E) Statistical chart of BMAL1 protein expression level at different time points. Data are expressed as mean ± SEM (*n* = 6 per group). *p < 0.05 compared with the control group

To explore the effect of APN deficiency on the expression of the *Bmal1* mRNA, we used real‐time PCR to detect the expression of *Bmal1* mRNA in the hippocampus of 4‐month‐old C57BL/6 mice and APN‐KO mice at CT4, CT8, CT12, CT16, CT20 and CT24. The results showed that the expression of *Bmal1* mRNA in the control group was relatively high at CT4, CT12, CT20 and CT24, with a peak at CT20, whilst the expression was relatively low at CT8 and CT16, with a trough at CT8. The rhythmic expression of *Bmal1* mRNA in APN‐KO mice was abnormal, showing relatively high expression at CT4, CT8, CT20 and CT24, with a peak at CT24, and relatively low expression at CT12 and CT16, with a trough at CT12. The *Bmal1* mRNA expression level at CT12 was significantly lower than that at CT12 in the control group (*p* < 0.05) (Figure 4A,B).

We then examined the expression of the BMAL1 protein. The data showed that the expression level of BMAL1 protein was the highest at CT24 in the control group, whilst it was the highest at CT4 in the APN‐KO group. Compared with that in the control group, the expression of BMAL1 protein in APN‐KO mice was significantly increased, which was statistically significant at CT4, CT12, CT16 and CT20 (*p* < 0.05) (Figure 4C‐E). These results suggest that APN deficiency could induce abnormal expression of the Bmal1 mRNA/protein in the hippocampus.

### 3.3 Adiponectin ameliorated abnormal expression of Bmal1 mRNA/protein induced by Aβ31‐35 in HT22 hippocampal neurons cells

**FIGURE 5:**
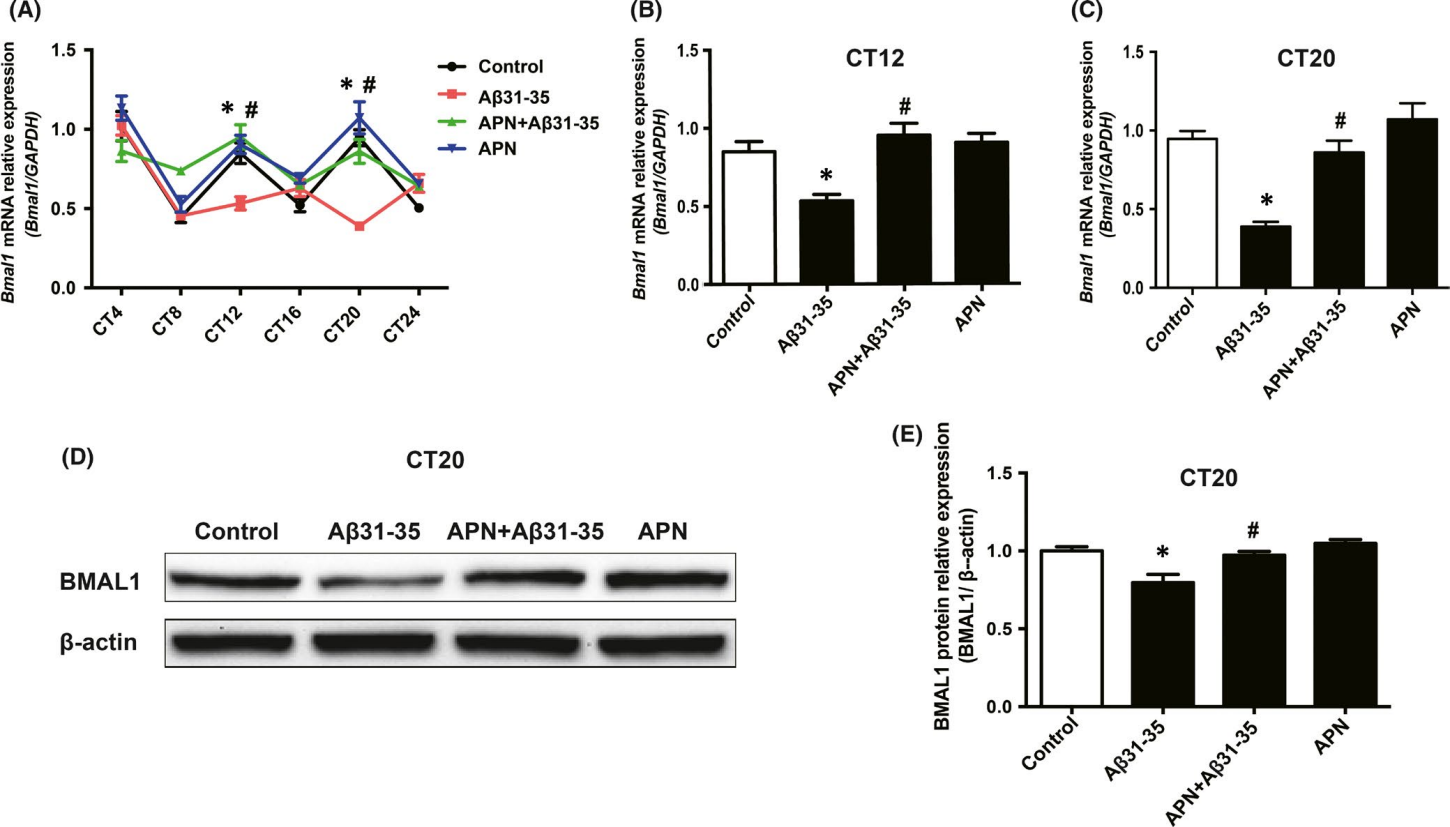
Effect of APN on Aβ31‐35‐induced abnormal expression of Bmal1 mRNA/protein in HT22 hippocampal cells. (A) Real‐time PCR was used to detect the mRNA expression of *Bmal1* in HT22 hippocampal cells at different time points in each group. (B, C) mRNA levels of *Bmal1* at CT12 and CT20 in each group. (D, E) Western blotting analysis showing the protein expression of BMAL1 at CT20. Data are expressed as the mean ± SEM (*n* = 6 per group). *p < 0.05 compared to the control group; ^#^ *p* < 0.05 compared to the Aβ31‐35 group

To explore whether APN could improve the abnormal expression of Bmal1 induced by Aβ31‐35, we identified *Bmal1* mRNA at CT4, CT8, CT12, CT16, CT20 and CT24 in HT22 hippocampal neurons after pretreatment with 10 ng/ml APN for 1 h by real‐time PCR. The results showed that the expression level of *Bmal1* mRNA was significantly higher at CT12 and CT20 after APN pretreatment compared with that in the Aβ31‐35 alone treatment group (*p* < 0.05), and the abnormal expression of *Bmal1* mRNA induced by Aβ31‐35 was partially reversed, whilst there was no significant difference in the levels of *Bmal1* mRNA expression between the APN alone and control groups (*p* > 0.05) (Figure 5A‐C). The expression of BMAL1 protein increased remarkably at CT20 after APN pretreatment for 1 h compared with that in the Aβ31‐35 treatment group (*p* < 0.05) (Figure 5D,E), suggesting that APN could ameliorate the abnormal expression of the Bmal1 mRNA/protein induced by Aβ31‐35 in HT22 cells.

### 3.4 Adiponectin could improve Aβ31‐35‐induced abnormal expression of Bmal1 mRNA/protein by inhibiting the activity of GSK3β

**FIGURE 6:**
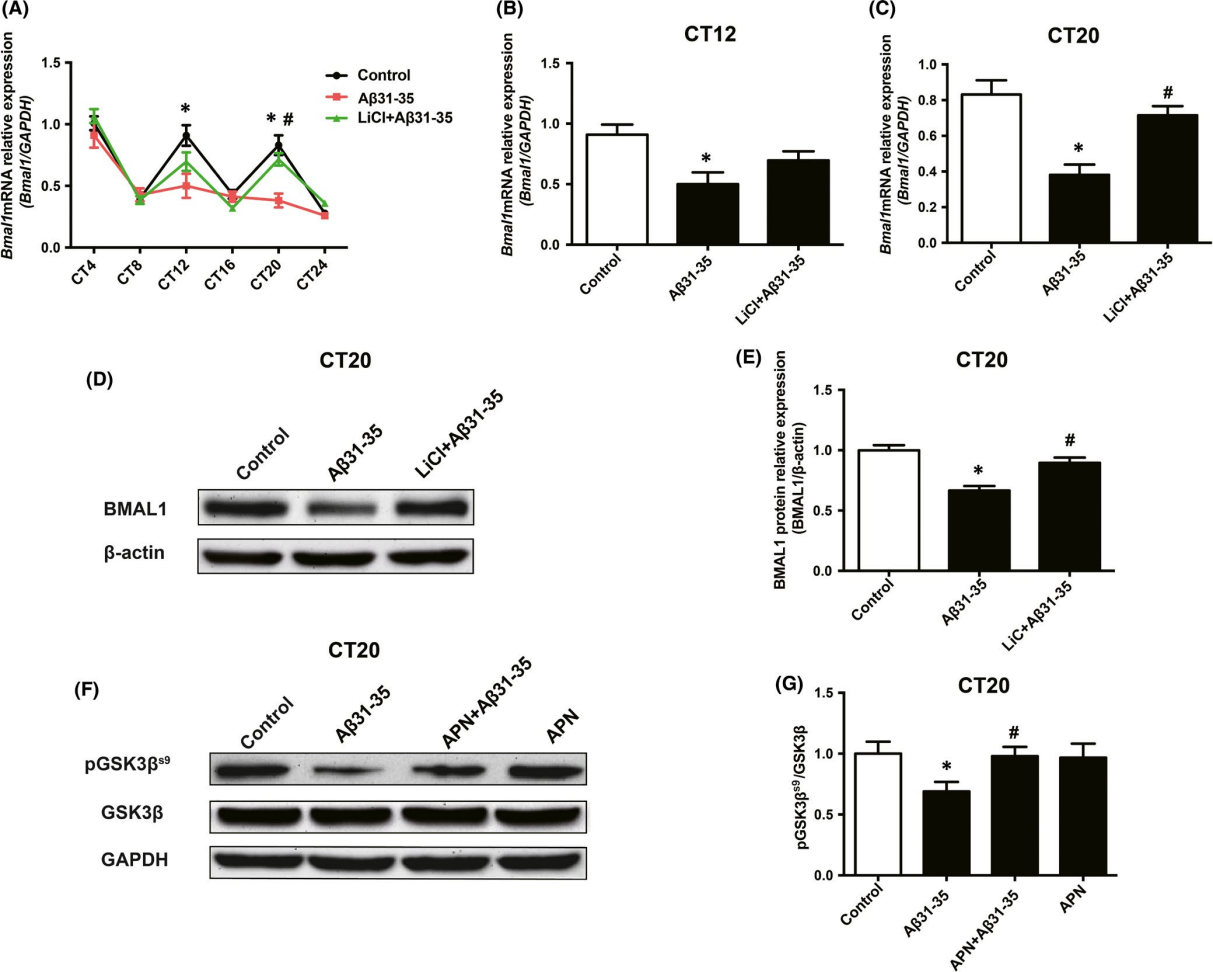
Adiponectin (APN) could improve Aβ31‐35‐induced abnormal Bmal1 mRNA/protein expression by inhibiting the activity of GSK3β. (A) Real‐time PCR was used to measure *Bmal1* mRNA expression in HT22 cells of the control group, Aβ31‐35 group, and LiCl +Aβ31‐35 group at different time points. (B, C) mRNA levels of *Bmal1* at CT12 and CT20 in each group. (D, E) Western blotting analysis showing the protein expression of BMAL1 at CT20. (F) The protein expression of PGSK3β^S9^ and GSK3β in the control group, Aβ31‐35 group, APN +Aβ31‐35 group and APN alone group was detected by western blotting. (G) Quantitative analysis of the PGSK3β^S9^/GSK3β ratio in each group. Data are expressed as the mean ± SEM (*n* = 6 per group). *p <0.05 compared to the control group; ^#^ *p* < 0.05 compared to the Aβ31‐35 group

To explore the role of GSK3β activity in APN improvement in Aβ31‐35‐induced abnormal expression of Bmal1, we used the GSK3β inhibitor LiCl to increase the ratio of pGSK3β^S9^/GSK3β and inhibit the activity of GSK3β. Then, the expression of *Bmal1* mRNA was detected at CT4, CT8, CT12, CT16, CT20 and CT24 after pretreatment with 30 μmol/L LiCl for 20 min. The results showed that the expression of *Bmal1* mRNA was significantly increased at CT20 after LiCl pretreatment compared with that in the Aβ31‐35 alone treatment group (*p* < 0.05), and the abnormal expression of *Bmal1* mRNA was partially ameliorated (Figure 6A‐C).

Furthermore, we selected the CT20 point to detect the protein expression of BMAL1 after LiCl pretreatment for 20 min. The data showed that the expression of BMAL1 protein was significantly higher in the LiCl pretreatment group than that in the Aβ31‐35 alone treatment group (*p* < 0.05) (Figure 6D,E), suggesting that the increase in GSK3β activity may be involved in the abnormal expression of BMAL1 protein induced by Aβ31‐35 in HT22 cells. Subsequently, we detected the expression of pGSK3β^S9^ and GSK3β proteins in HT22 cells after pretreatment with APN. The results showed that the increased GSK3β activity induced by Aβ31‐35 was effectively reversed after pretreatment with 10 ng/ml APN for 1 h, which was shown by an obvious increase in the expression of pGSK3β^S9^ (Figure 6F) and the ratio of pGSK3β^S9^/GSK3β (*p* < 0.05) (Figure 6F,G), indicating that APN could reverse the increased GSK3β activity caused by Aβ31‐35 in HT22 cells. Collectively, these results suggest that APN may ameliorate the Aβ31‐35‐induced abnormal expression of the Bmal1 mRNA/protein by inhibiting the activity of GSK3β.

## 4 DISCUSSION

In the present study, APN‐KO mice showed Aβ deposition, circadian rhythm disturbance, and abnormal expression of the Bmal1 mRNA/protein. APN pretreatment improved the abnormal expression of the Bmal1 mRNA/protein induced by Aβ 31‐35 in vitro. In addition, Aβ31‐35 induced an increase in GSK3β activity in HT22 cells. When GSK3β activity was inhibited, Aβ31‐35‐induced abnormal expression of the Bmal1 mRNA/protein was significantly ameliorated, and APN reversed the increased activity of GSK3β. Therefore, APN can improve Aβ31‐35‐induced abnormal expression of Bmal1 mRNA/protein by inhibiting the activity of GSK3β.

Alzheimer's disease is the most common cause of dementia worldwide, and it is an age‐related neurodegenerative disease.^20^ Typical pathological features of AD include senile plaques formed by the deposition of Aβ, neurofibrillary tangles formed by hyperphosphorylated tau protein, and a considerable loss of neurons,^21^, ^22^ amongst that, Aβ accumulation plays a vital role in the development of AD.^23^

Studies have shown that circadian rhythm disorder often occurs early on in AD, with sleep‐wake cycle disruption and night sleep fragmentation. The triple‐transgenic mouse model of AD, at the age of 3 months, exhibited a significant circadian rhythm disorder with more daytime activity and less night‐time activity.^24^ Studies have shown that circadian rhythm disorders are associated with the subsequent development of a series of symptoms such as decreased learning and memory ability and cognitive impairment.^25^, ^26^ Furthermore, circadian rhythm disorders are associated with abnormal Aβ deposition in the brain.^27^ Aβ31‐35 has only five amino acids and has been thought to be a main active centre of Aβ neurotoxicity. The lower molecular weight and the low aggregation ability permit Aβ31‐35 to rapidly enter the cells and exert neurotoxic effects.^28^, ^29^ Our previous research also found that Aβ31‐35, as one of the toxic core fragments of Aβ, could induce obvious circadian rhythm disorder (when administered via intrahippocampal injection) in C57BL/6 mice, and this disorder manifested in the form of an unclear movement phase and resting phase, as well as a prolonged free‐running period.^4^ However, there are no effective prevention and treatment measures for Aβ‐induced circadian rhythm disorders.

Several studies have found a potential relationship between AD and T2DM. In T2DM patients, the grey matter content of the frontotemporal area and the volume of the hippocampus decreased. The risk of cognitive impairment and development of AD in T2DM patients is 1.5 to 2 times higher than that in patients without T2DM.^30^, ^31^ Meanwhile, approximately 80% of the AD patients have T2DM or impaired glucose tolerance.^32^ Insulin resistance is a common pathophysiological feature of AD and T2DM. Insulin resistance is a reduced sensitivity of body tissues to insulin, which is one of the earliest and most significant metabolic defects in T2DM.^33^ Similar insulin response defects have also been observed in AD patients and animal models.^31^, ^34^ In addition, neuronal insulin signalling is closely associated with Aβ deposition, tau protein phosphorylation, synaptic plasticity and memory function.^35^, ^36^ Impaired insulin signalling and insulin resistance play a vital role in pathological changes in AD. Studies have shown that APN can increase insulin sensitivity. APN is an adipocyte‐derived hormone that is secreted into the circulatory system. As an endocrine hormone, APN plays a variety of physiological roles, including regulating glucose and lipid metabolism and anti‐inflammatory and antioxidant effects.^37^ Furthermore, APN can also act as an insulin sensitizer to induce insulin sensitization by activating the insulin receptor signalling pathway.^9^ Clinical studies have found that both patients with mild cognitive impairment and patients with AD have decreased levels of circulating APN.^10^ Other studies have revealed that low molecular weight APN (trimers and hexamers) can be detected in human cerebrospinal fluid (CSF), indicating that APN can cross the blood‐brain barrier (BBB) to enter the central nervous system. APN exerts its biological effects by binding to adiponectin receptors (AdipoR1 and AdipoR2) and APN receptors are abundantly expressed in the hippocampus.^11^ Various AD‐like pathological changes can be observed in APN‐KO mice and AdipoR1‐deficient mice, including Aβ deposition, tau protein hyperphosphorylation, neuroinflammation, synapse loss and neuronal apoptosis.^11^, ^12^ In this study, we found abnormal deposition of Aβ in the hippocampus of APN‐KO mice, suggesting that APN deficiency is closely related to the development of AD. In contrast, the relationship between APN deficiency and circadian rhythm disorder in AD is still unclear. In this study, we investigated the circadian rhythm of APN‐KO mice using wheel‐running behaviour experiments. The results showed that APN‐KO mice had circadian rhythm disorder, a prolonged free‐running cycle, an increased subjective daytime activity and an increased ratio of subjective daytime activity to total activity compared with the mice in the control group, and this implies that APN deficiency could induce circadian rhythm disorders.

The circadian rhythm is the continuous fluctuation of various physiological activities in an approximately 24‐h cycle that depends on the transcriptional‐translational feedback loop composed of a series of clock genes and proteins, amongst that, Bmal1 is an important positive regulator. CLOCK‐BMAL1 heterodimer activates the transcription of *Per* and *Cry* genes by binding to E‐box enhancers. The translated PER and CRY proteins translocate into the nucleus, where they act as negative regulators by inhibiting the transcriptional activation of the CLOCK‐BMAL1 heterodimer. Furthermore, the CLOCK/BMAL1 heterodimer promotes the transcription of *Rev*‐*erbα*, and the REV‐ERBα proteins repress *Bmal1* transcription by competing with RORα binding to the RORE motif in the *Bmal1* promoter. Through this feedback loop, the CLOCK/BMAL1 heterodimer initiates the transcription of circadian clock genes and clock‐controlled genes to form a rhythmic cycle, resulting in the transcription of *Bmal1* presenting rhythmic oscillations.^38^, ^39^ It can be seen that the existence and regular periodic oscillation of Bmal1 play a vital role in maintaining the circadian rhythm. However, whether adiponectin deficiency can induce the abnormal expression of BMAL1 is unclear. In this study, we first detected the expression of *Bmal1* mRNA in the hippocampus of APN‐KO mice using real‐time PCR, and the results showed that the expression level of *Bmal1* mRNA was significantly lower than that in the control group at CT12. The expression of BMAL1 protein was further detected by western blotting, and it was found that the expression of BMAL1 protein increased at CT4, CT12, CT16 and CT20, which was statistically significant when compared to that in the control group. These results suggest that the Bmal1 mRNA/protein is abnormally expressed in the hippocampus of APN‐KO mice. The decrease in *Bmal1* mRNA expression and the increase in BMAL1 protein expression in APN‐KO mice may be related to lipid metabolism. Evidence has shown that the BMAL1 protein is involved in adipogenesis. BMAL1 promotes *Rev*‐*erbα* transcription, and Rev‐erbα contributes to adipogenesis by enhancing the expression of adipocyte differentiation‐related factors aP2 and C/EBPα.^40^, ^41^ Some studies have found that BMAL1 protein expression is increased in the suprachiasmatic nucleus of high‐fat diet‐induced obese mice.^42^ APN participates in lipid catabolism, which promotes fatty acid oxidation, that is, the level of APN is negatively correlated with the fat content.^43^ In this study, we found that the expression level of the BMAL1 protein in the hippocampus of APN‐KO mice increased significantly at CT4, CT12, CT16 and CT20, which is consistent with the findings of previous studies. The decrease in the expression of *Bmal1* mRNA at CT12 may be related to feedback inhibition of protein aggregation on mRNA.

The above results confirmed that APN deficiency induces AD‐like pathological changes, circadian rhythm disorders, and abnormal expression of the core circadian clock gene *Bmal1*. Other studies have shown that APN reduces the AD‐like pathological characteristics of APP/PS1 mice by activating APN signaling,^13^ suggesting that APN has potential therapeutic significance in AD. However, whether APN can improve circadian rhythm disorder in AD remains to be determined. Studies have reported that APN plays an important role in the proliferation and neuroprotection of hippocampal nerve cells.^44^, ^45^ AdipoRon (as an agonist of adiponectin) can alleviate the cognitive dysfunction of AD mice, inhibit Aβ deposition and promoted the impaired hippocampal NSCs proliferation on the early stage in vivo.^46^ Meanwhile, the hippocampus is the primary lesion of AD and has its own circadian rhythm, we used HT22 hippocampal nerve cells to verify the above hypothesis in vitro. The results showed that the expression of *Bmal1* mRNA in HT22 cells was relatively high at CT4, CT12 and CT20 and low at CT8, CT16 and CT24. The expression of *Bmal1* mRNA was abnormal after treatment with Aβ31‐35, showing that the expression decreased at CT12 and CT20, and the decrease at CT20 was the most significant. *Bmal1* mRNA shows a normal 24‐h cyclical rhythm. After Aβ31‐35 treatment, the expression of *Bmal1* mRNA fluctuated and the circadian rhythm was disturbed. Rhythm data have several basic characteristics.^47^ When the circadian rhythm is disturbed, the rhythm indicators such as periodicity, phase and amplitude will alter, leading to significant changes in *Bmal1* mRNA at some CTs, but no significant difference at some CTs. We hypothesized that the *Bmal1* mRNA expression abnormalities at different CTs appear to be selective after Aβ31‐35 treatment. The expression of the BMAL1 protein at CT20 was consistent with that at the gene level. We found that APN can reverse the abnormal expression of the Bmal1 mRNA/protein induced by Aβ31‐35. Compared with Aβ31‐35 alone treatment, the expression of *Bmal1* mRNA after APN pretreatment was increased significantly at CT12 and CT20, and the expression of the BMAL1 protein at CT20 was consistent with that at the gene level.

The crosstalk between the circadian clock and metabolism is essential for maintaining metabolic homeostasis.^19^ There are many factors that affect the expression of BMAL1 protein, such as lipid metabolism, energy metabolism and oxidative stress. Evidence has shown that the BMAL1 protein is involved in adipogenesis and APN participates in lipid catabolism, which promotes fatty acid oxidation. In this study, we found that the expression level of the BMAL1 protein in the hippocampus of APN‐KO mice increased significantly at CT4, CT12, CT16 and CT20, However, Studies have shown that BMAL1 protein was attenuated in 5XFAD cortex,^48^ whilst the protein levels of BMAL1 are significantly elevated in impaired astrocytes of cerebral cortex from patients with AD.^49^ We speculate that the increase or decrease of BMAL1 protein expression can affect the pathological development of AD, and the rhythmic expression of BMAL1 protein plays a vital role in maintaining the normal circadian rhythm. Studies have found that Aβ induced the degradation of BMAL1 protein and impacted on the metabolic stability BMAL1 protein.^3^ In this study, Aβ31‐35 induced a decrease in BMAL1 protein expression, and the rhythmic expression of BMAL1 was disturbed. APN can improve the abnormal expression of BMAL1 protein caused by Aβ in hippocampal cells. APN is a protein that regulates various metabolic diseases.^50^ The crosstalk between APN and the biological clock maintains the metabolic homeostasis of the cell. Therefore, the expression of BMAL1 protein is too high or too low; it means that its own circadian rhythm is destroyed, and APN can maintain its normal stability. However, the mechanism by which APN ameliorates Aβ31‐35‐induced abnormal expression of Bmal1 mRNA/protein is still unclear.

Increasing evidence has suggested that GSK3β plays an important role in the maintenance of circadian rhythm, and the change in its activity is closely related to the regulation of Bmal1. A key feature of GSK3β is that it is active in its default state and that it is inactivated by phosphorylation Ser‐9 for GSK3β (pGSK3β^S9^). The ratio of pGSK3β^S9^ to GSK3β is an important indicator of GSK3β activity. When the level of pGSK3β^S9^ decreased and the ratio of pGSK3β^S9^ to GSK3β decreased, the activity of GSK3β increased; otherwise, the activity was inhibited.^51^, ^52^ The increase in GSK3β activity can destroy the circadian rhythm of BMAL1 protein expression,^51^ whilst the inhibition of GSK3β activity can enhance the stability of BMAL1 protein and increase its expression level.^15^ In addition, GSK3β phosphorylates and stabilizes the orphan nuclear receptor REV‐ERBα, a negative component of the circadian clock. Inhibition of GSK3β activity leads to the degradation of REV‐ERBα and activates *Bmal1* transcription.^53^ Therefore, GSK3β is critical for rhythmic Bmal1 expression. Another study has shown that Aβ can induce an increase in GSK3β activity.^16^ In this study, we also found that GSK3β activity increased significantly after treatment with 5 μmol/L Aβ31‐35 in HT22 cells. Next, we investigated whether GSK3β activated by Aβ31‐35 is involved in Aβ31‐35‐induced abnormal expression of Bmal1 mRNA/protein. In the present study, the abnormal expression of *Bmal1* mRNA in HT22 cells caused by Aβ31‐35 was ameliorated after pretreatment with LiCl, a specific inhibitor of GSK3β, especially at CT20, which is similar to what was observed for BMAL1 protein expression, suggested that the increase in GSK3β activity contributed to the abnormal expression of Bmal1 mRNA/protein.

Many studies have shown that APN can inhibit the activity of GSK3β. APN attenuated streptozotocin‐induced tau hyperphosphorylation and cognitive deficits by rescuing the PI3K/Akt/GSK‐3β pathway in rats.^54^ To investigate whether Aβ31‐35 deposit increases GSK3β activity, we further measured the protein level of both GSK3β in HT22 cell lines. We found that APN could significantly reverse the increase in GSK3β activity induced by Aβ31‐35. Considering the key role of increased GSK3β activity in the abnormal expression of Bmal1 mRNA/protein induced by Aβ31‐35 and the fact that APN could ameliorate the abnormal expression of Bmal1 induced by Aβ31‐35, we suggest that APN may improve Aβ31‐35‐induced abnormal expression of Bmal1 by inhibiting the activity of GSK3β.

In summary, our study revealed that APN deficiency could induce circadian rhythm disorder and showed that APN might improve the abnormal expression of Bmal1 mRNA/protein induced by Aβ31‐35 in HT22 hippocampal neurons by inhibiting the activity of GSK3β, which provides a novel approach for the treatment of AD.

## CONFLICT OF INTEREST

The authors confirm that there are no conflicts of interest.

## AUTHOR CONTRIBUTIONS

**Yuan Yuan:** Investigation (equal); Writing‐original draft (equal). **Chen Li:** Investigation (equal). **Shuai Guo:** Formal analysis (equal); Writing‐original draft (equal). **Cong Sun:** Investigation (equal). **Na Ning:** Investigation (equal). **Haihu Hao:** Investigation (equal). **Li Wang:** Investigation (equal). **Yunfei Bian:** Resources (equal). **Huirong Liu:** Resources (equal). **Xiaohui Wang:** Conceptualization (equal); Funding acquisition (equal); Resources (equal); Writing‐review & editing (equal).

## Supporting information

Figure S1

## Data Availability

The data that support the findings of this study are available from the corresponding author upon reasonable request.
