# Supplementary figures and images for: Adiponectin improves amyloid‐β 31‐35‐induced circadian rhythm disorder in mice
Source: J Cell Mol Med. 2021 Sep 15;25(20):9851–62. doi: 10.1111/jcmm.16932 (PMC8505833; doi:10.1111/jcmm.16932)

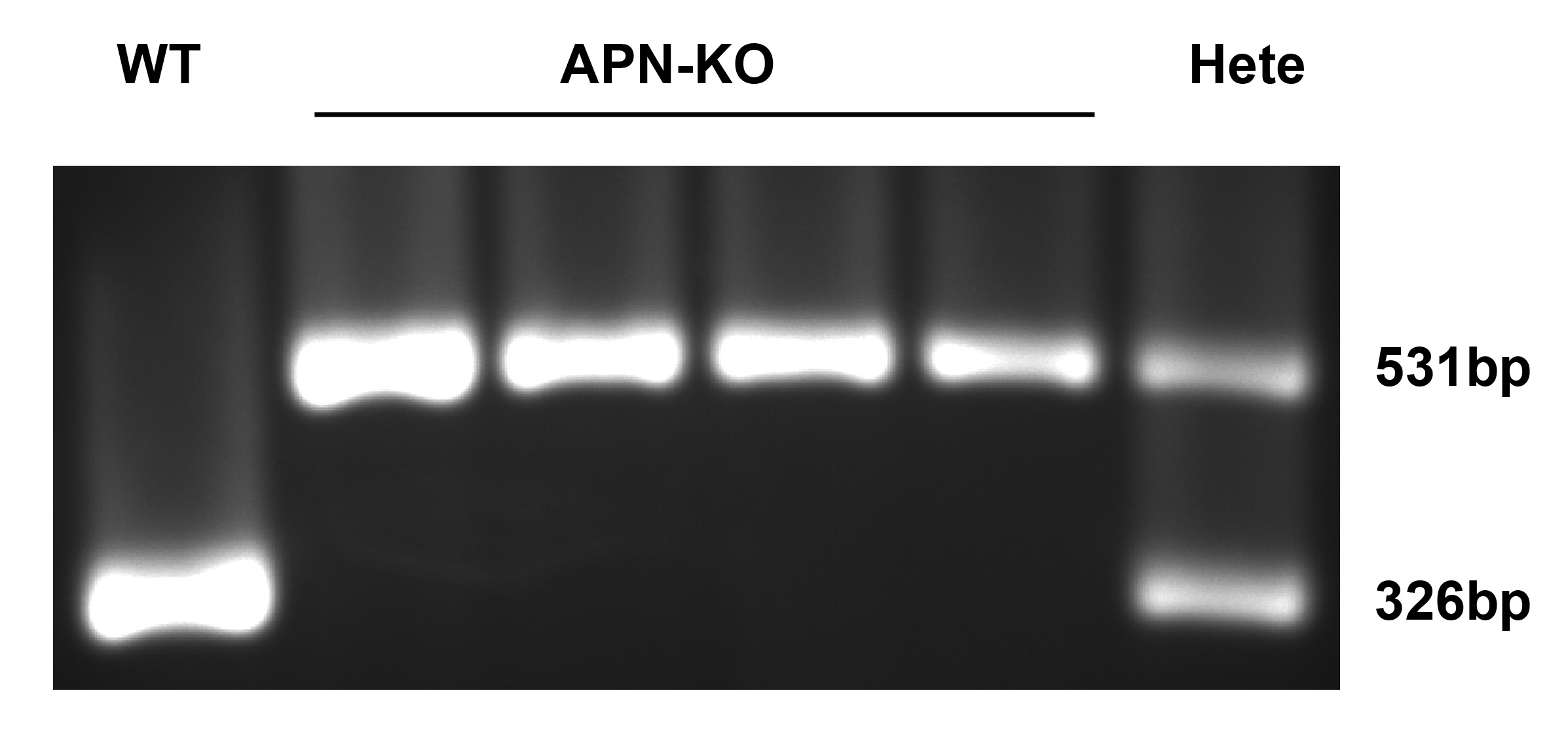

Supplement: Supplementary file 1 — Figure S1 [file JCMM-25-9851-s001.jpg]
